# A fragment of the alarmin prothymosin α as a novel biomarker in murine models of bacteria-induced sepsis

**DOI:** 10.18632/oncotarget.18149

**Published:** 2017-05-24

**Authors:** Pinelopi Samara, Vivi Miriagou, Michael Zachariadis, Olga Mavrofrydi, Vasilis J. Promponas, Skarlatos G. Dedos, Panagiota Papazafiri, Hubert Kalbacher, Wolfgang Voelter, Ourania Tsitsilonis

**Affiliations:** ^1^ Department of Biology, National and Kapodistrian University of Athens, Athens, Greece; ^2^ Laboratory of Bacteriology, Hellenic Pasteur Institute, Athens, Greece; ^3^ Institute of Biosciences and Applications, National Center for Scientific Research “Demokritos”, Agia Paraskevi, Athens, Greece; ^4^ Bioinformatics Research Laboratory, Department of Biological Sciences, University of Cyprus, Nicosia, Cyprus; ^5^ Interfaculty Institute of Biochemistry, University of Tübingen, Tübingen, Germany

**Keywords:** apoptosis, biomarker, Klebsiella pneumoniae, proTα(100-109), sepsis, Immunology and Microbiology Section, Immune response, Immunity

## Abstract

Sepsis is a life-threatening condition that requires urgent care. Thus, the identification of specific and sensitive biomarkers for its early diagnosis and management are of clinical importance. The alarmin prothymosin alpha (proTα) and its decapeptide proTα(100-109) are immunostimulatory peptides related to cell death. In this study, we generated bacterial models of sepsis in mice using two *Klebsiella pneumoniae* strains (L-78 and ATCC 43816) and monitored sepsis progression using proTα(100-109) as a biomarker. Serum concentration of proTα(100-109) gradually increased as sepsis progressed in mice infected with L-78, a strain which, unlike ATCC 43816, was phagocytosed by monocytes/macrophages. Analysis of splenocytes from L-78-infected animals revealed that post-infection spleen monocytes/macrophages were gradually driven to caspase-3-mediated apoptosis. These results were verified *in vitro* in L-78-infected human monocytes/macrophages. Efficient phagocytosis of L-78 by monocytes stimulated their apoptosis and the concentration of proTα(100-109) in culture supernatants increased. Human macrophages strongly phagocytosed L-78, but resisted cell death. This is the first report suggesting that high levels of proTα(100-109) correlate, both *in vitro* and *in vivo*, with increased percentages of cell apoptosis. Moreover, we showed that low levels of proTα(100-109) early post-infection likely correlate with sepsis resolution and thus, the decapeptide could eventually serve as an early surrogate biomarker for predicting bacteria-induced sepsis outcome.

## INTRODUCTION

Sepsis (from Greek σῆψις, decay) is a life-threatening response to infection, leading to excessive tissue damage, organ failure, and death. It is associated with factors related to the invading pathogens, most commonly Gram-negative bacteria, as well as the status of the host's immune system [1]. Excessive immune cell activation leads to release of inflammatory mediators, determined usually in blood samples and used as biomarkers of infection.

Established sepsis biomarkers include cytokines (e.g. interleukin 6 and tumor necrosis factor alpha [TNF-α]), lactate, the acute-phase C-reactive protein, the pro-hormone procalcitonin and damage-associated molecular patterns (DAMPs)/cell surface receptors (e.g. triggering receptor expressed on myeloid cells-1) [2]. Among DAMPs, the prototype alarmin high-mobility group box 1 protein (HMGB1) has been extensively studied both as a biomarker and a therapeutic target [3]. Nevertheless, sepsis diagnosis and assessment of its severity is rather complex because of its heterogeneity and the lack of sensitive and specific assays discriminating infectious from non-infectious cases. Consequently, novel surrogate biomarkers are needed.

Experimental animal studies are essential in identifying and characterizing the pathophysiological stages of sepsis and for developing new therapeutic strategies and diagnostic tools. Bacterial infusion in mice is the most widely used model, as introduction of a single pathogen under controlled conditions provides reproducibility of the infection [4].

Our research group studies the immune-related mechanisms of action of the polypeptide prothymosin alpha (proTα) and its immunologically active fragment proΤα(100-109) [5]. ProΤα(100-109) is generated upon truncation of proTα by activated caspase-3, in cells undergoing apoptosis [6]. Both molecules extracellularly act as alarmins, ligate TLR-4, induce the maturation of dendritic cells, and mount T_H_1-biased immune responses *in vitro* [7–9]. We hypothesized that if proTα and proΤα(100-109) are released from damaged cells, they could serve as immune stimuli, acting similarly to HMGB1 [10]. To detect and quantify the extracellular release of these peptides, we previously established a highly sensitive ELISA for proTα(100-109) and induced massive cell death in mice, by infecting them with *Streptococcus pyogenes* (*S. pyogenes*). We showed that the concentration of proTα(100-109) in serum of *S. pyogenes*-infected mice was significantly increased during the progress of infection [11].

To further evaluate the role of proTα(100-109) as a sepsis biomarker, we used *Klebsiella pneumoniae* (*K. pneumoniae*) as challenge-microorganism, a Gram-negative bacterium associated with aggressive infections (e.g. septicemia and pneumonia) and numerous nosocomial outbreaks [12]. We selected two *K. pneumoniae* strains of diverse virulence and properties [13]. We analyzed *K. pneumoniae* infection in mice and in human cells *in vitro*, focusing on the mechanisms *via* which these bacteria kill innate immune cells and lead to the generation of proTα(100-109). We further validated our results in a “moderate” model of sepsis in mice, and showed that early post-infection (pi) proTα(100-109) serum levels can predict mortality of individual mice infected with *K. pneumoniae*.

## RESULTS

### Low serum levels of proTα(100-109) were detected early post-infection with L-78

**Figure 1:**
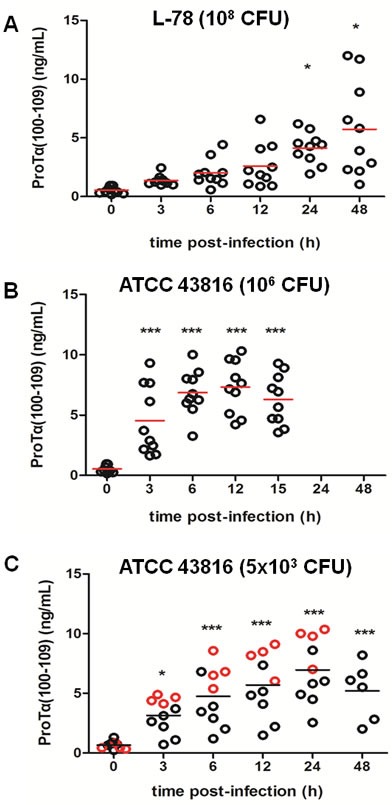
Concentration of proTα(100-109) in serum of mice during *Klebsiella pneumoniae* infection CD-1 mice were intraperitoneally infected with L-78 **A**. and ATCC 43816 **B**., **C**., respectively. Serum samples were collected prior to infection (0 h), at 3, 6, 12, 24, and 48 h post-infection (pi) for the L-78 and the 5×10^3^ CFU ATCC 43816 groups **A**. and **C**., and at 3, 6, 12, and 15 h pi for the 10^6^ CFU ATCC 43816 group **B**.. Each circle represents the concentration of proTα(100-109) from a single mouse at the corresponding time point. In C, red cirles mark mice that died between 30-45 h pi. Horizontal bars indicate mean values. *, *p* < 0.05; ***, *p* < 0.001 after one-way ANOVA followed by Dunnett's test (*vs* 0 h).

Animals were infected with the clinically isolated strain L-78, which is of low virulence (50% lethal dose [LD_50_] > 10^8^ colony-forming units [CFU]), highly resistant to antibiotics and endocytosed by monocytes/macrophages, and with the ATCC 43816 strain, which is highly infective (LD_50_ = 5 × 10^3^ CFU), sensitive to antibiotics and not endocytosed. Based on the Kaplan-Meier survival curves of Tzouvelekis [13] for immunocompetent mice, animals were intraperitoneally (ip) infected with 10^8^ CFU of L-78, and 5×10^3^ or 10^6^ CFU of ATCC 43816. Blood samples were collected at the time points shown in Figure 1. Mouse sera were analyzed by our ELISA for proTα(100-109) [11]. To rule out obvious non-specific interactions between proTα(100-109)-specific Abs and any cross-reacting substance present in murine serum, we assessed if the primary structure of the decapeptide can be depicted within the amino acid sequence of currently known mouse proteins or proteins known to be encoded in *K. pneumoniae* (Supplementary Table S1). A scan with the regular expression pattern against the proteins encoded in the most current version of the mouse genome showed that cross-reactivity was unlikely to occur.

In animals infected with 10^8^ CFU of L-78, serum concentration of proTα(100-109) gradually increased during the course of infection with maximum quantity (5.72 ng/mL) detected at 48 h pi (Figure 1A), which further reduced to background levels at 96 h (data not shown). Mice infected with 10^6^ CFU of ATCC 43816 exhibited a sharp increase in serum concentration of proTα(100-109) already in the first three hours pi (4.53 ng/mL) which remained relatively constant up to 15 h pi (6.29 ng/mL) (Figure 1B). The distinct patterns of rise in the levels of proTα(100-109) among the two murine groups could be attributed to the diverse virulence of the strains. Specifically, animals injected with L-78 showed transient signs of infection and all resisted infection and recovered within 48 h, while animals administered 10^6^ CFU of ATCC 43816 died at 15 h pi, due to generalized sepsis. Mice infected with 5×10^3^ CFU of ATCC 43816 (“moderate” model of sepsis), showed a different pattern of increase in serum proTα(100-109) levels, with some animals showing high and others low concentrations of the decapeptide early pi (Figure 1C). This group of mice was used to validate our results and data are further analyzed and discussed. To evaluate the impact of traumatic injections on proTα(100-109) levels, we additionally collected serum from mice punctured ip and injected with saline. No differences in proTα(100-109) levels prior and 3 h after traumatic injection were observed (data not shown).

### Spleen bacterial load increased in ATCC 43816-infected mice

At the same time points to blood collection, spleens from two mice of the L-78 and 10^6^ CFU ATCC 43816 groups were removed and bacterial load, expressed in CFU, was determined. Spleens were not contaminated with intestinal or other external bacteria during isolation, and the individual colonies on the plates were typical for *Klebsiella*. We sacrificed only two mice per time point per model based on the 3R principle (Replacement, Reduction, Refinement) for laboratory animal use, to verify similar results available in our laboratory from previous experiments.

**Figure 2:**
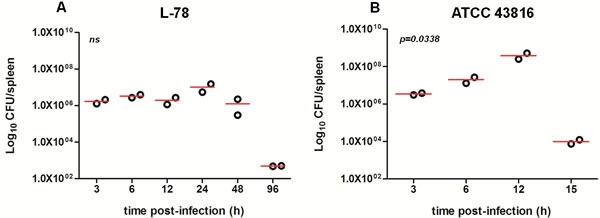
Bacterial load in the spleen of CD-1 mice infected with 10^8^ CFU L-78 A. and 10^6^ CFU ATCC 43816 B Two mice from each group were sacrificed at the same time points as in Figures 1A and 1B and for an additional time point (96 h) for L-78 infected animals to confirm bacterial clearance. Spleen homogenates were plated on agar and CFU counts were determined. Data are Log_10_CFU/spleen from two independent experiments. ns, not significant after one-way ANOVA.

It is well documented that ip administration of bacteria leads to rapid and effective delivery to the bloodstream resulting in systemic infection [14]. In animals infected with 10^8^ CFU of L-78 (0 h), the number of bacteria that entered the spleen between 3-12 h was approximately 10^6^ CFU, increased to ∼10^7^ CFU at 24 h and decreased thereafter (Figure 2A). In contrast, in animals that were infected with 10^6^ CFU of ATCC 43816, the bacterial load in spleen significantly increased between 6-12 h (up to ∼7×10^8^ CFU at 12 h) and sharply decreased afterward, as animals died due to generalized sepsis at 15 h (*p* = 0.0338; Figure 2B). Apparently, ATCC 43816, although injected at a lower inoculum, multiplied rapidly *in vivo*, and led to multi-organ failure within 15 h pi. At this time point, generalized sepsis was accompanied by extensive necrosis of vital internal organs (including spleen), and lower bacterial spleen load was detected (∼10^4^ at 15 h).

### L-78 infection induced apoptosis and ATCC 43816 necrosis in mouse spleen cells

**Figure 3:**
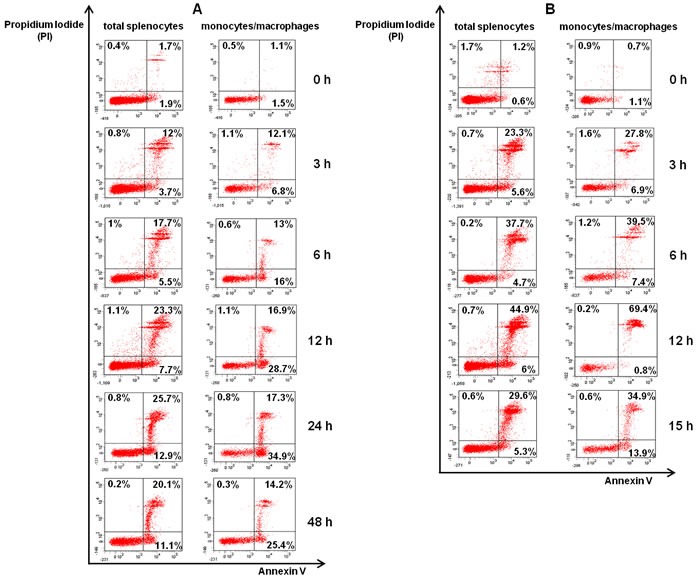
Spleen monocytes/macrophages infected with L-78 were driven to apoptosis Total splenocytes and spleen monocytes/macrophages isolated from *K. pneumoniae* infected-mice at the indicated time points post-infection (0-48 h for L-78 **A.** and 0-15 h for ATCC 43816 **B.**) were stained with Annexin V/PI and analyzed by flow cytometry. Percentages in lower right quadrants are apoptotic cells (Annexin V+PI-), and in upper quadrants necrotic cells (PI+). Dot plots shown are from one representative animal out of two independently analyzed with similar results.

The type and extent of cell death in spleen cells were evaluated by flow cytometry, using Annexin V and propidium iodide (PI) staining. We analyzed total splenocytes, as well as spleen monocytes/macrophages (CD11b+) (Supplementary Figure S1). As shown in Figure 3, prior to infection (0 h), few mouse splenocytes were apoptotic (∼2% Annexin V+PI-). During the course of L-78 infection (3-48 h), percentages of apoptotic splenocytes gradually increased (up to 12.9%) (Figure 3A, left panel). This was better shown in spleen monocytes/macrophages, where percentages of apoptotic cells significantly increased up to 24 h (34.9%) and then decreased (Figure 3A, right panel). In animals infected with 10^6^ CFU of ATCC 43816, the percentages of apoptotic total splenocytes were low and remained unaltered (Figure 3B, left panel), but necrotic splenocytes significantly increased from 3 to 12 h pi (Figure 3B, right panel). High percentages of necrotic monocytes/macrophages particularly at 12 h pi (69.6%) were also detected.

Combining the ELISA values (Figure 1A) with the results of flow cytometry (Figure 3A) in L-78-infected mice, a positive correlation between serum proTα(100-109) concentration and the percentage of apoptotic splenic monocytes/macrophages was observed. Before infection both were low, whereas pi the levels of the decapeptide gradually increased following the increase in the percentage of apoptotic cells.

### Procaspase-3 was cleaved early during the course of L-78 infection

**Figure 4:**
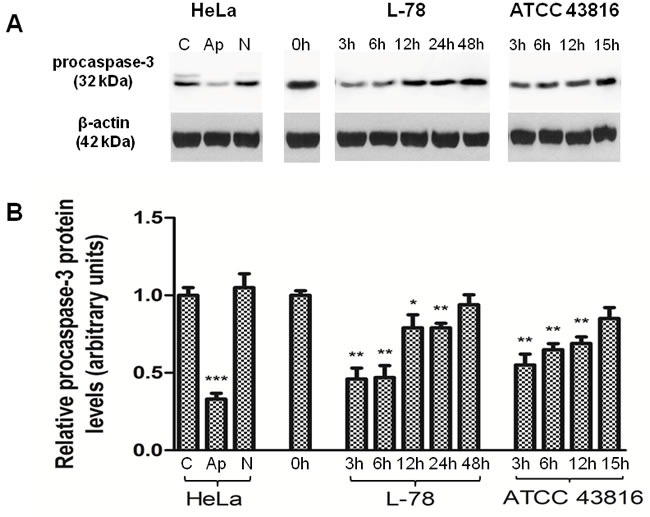
Procaspase-3 levels decreased early after L-78 infection in murine spleen lysates **A**. Total protein extracts of pooled splenocytes from L-78- and ATCC 43816-infected mice were immunoblotted against procaspase-3. Apoptotic and necrotic HeLa cells were used as controls. β-Actin was used as a loading control. All samples were run on and cut from the same gel. **B**. Densitometric analysis of procaspase-3. Data are means ± SD from three independent experiments. C, HeLa cells incubated in medium; Ap, apoptotic HeLa cells; N, necrotic HeLa cells. *, *p* < 0.05; **, p < 0.01; ***, p < 0.001, compared with control after Student's unpaired *t*-test.

To verify activation of the apoptotic pathway during *K. pneumoniae* infection, we determined procaspase-3 levels in lysates of mouse splenocytes by Western blotting. For controls, we treated HeLa cells with TNF-α and emetine which led the majority of cells to apoptosis, or high concentration of doxorubicin which induced massive necrosis (Supplementary Figure S2). Western blot analysis showed that the levels of procaspase-3 in HeLa lysates were significantly reduced only under apoptotic conditions (*p* = 0.001), and remained unaltered when spleen cells were driven to necrosis (Figure 4).

In our *in vivo* models, early after infection (3 and 6 h) with L-78, the relative level of procaspase-3 was reduced by half compared to pre-infection levels (*p* = 0.0046 and *p* = 0.0056, respectively) and was restored to background levels at 12-24 h pi in parallel with bacterial clearance (*p* = 0.0407 at 12 h; *p* = 0.0061 at 24 h). In mice infected with 10^6^ CFU of ATCC 43816, procaspase-3 protein levels decreased early pi (*p* = 0.0066 at 3 h) and progressively increased from 6 to 15 h (*p* = 0.0037 at 6 h; *p* = 0.0055 at 12 h).

### ProTα(100-109) levels increased only in culture supernatants of L-78-infected human monocytes led to apoptosis

**Figure 5:**
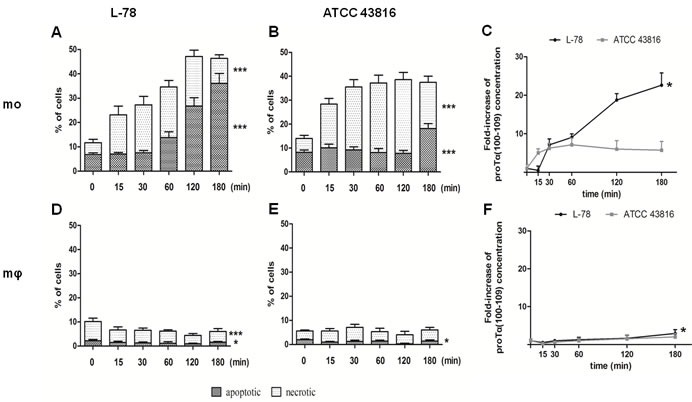
High levels of proTα(100-109) correlated with higher percentages of apoptotic human monocytes *in vitro* infected with L-78 Human monocytes **A**. and **B**. and *in vitro*-differentiated macrophages **D**. and **E**. infected with L-78 **A**. and **D**. or ATCC 43816 **B**. and **E**. were harvested at 0-180 min, stained with Annexin V/PI and analyzed by flow cytometry. Dark columns show percentages of apoptotic cells; white columns, necrotic cells. Mean values ± SD from 5 different healthy blood donors are shown. ***, *p* < 0.0001 for L-78-infected apoptotic and necrotic monocytes **A**., for ATCC 43816-infected apoptotic and necrotic monocytes **B**. and for L-78-infected necrotic macrophages **D**.. *, *p* = 0.0106 for L-78-infected apoptotic macrophages **D**. and *p* = 0.0229 for ATCC 43816-infected apoptotic macrophages **E**. (one-way ANOVA). Supernatants from the same cultures were analyzed by ELISA and proTα(100-109) concentration is expressed as mean fold-increase ± SD from three independent experiments **C**. and **F**. *, *p* = 0.0015 for L-78-infected monocytes **C**. and *p* = 0.0324 for L-78-infected macrophage **F**. (one-way ANOVA).

Human monocytes and macrophages co-cultured with L-78 or ATCC 43816 were recovered at 0-180 min and analyzed by flow cytometry, following Annexin V/PI staining. Monocytes infected with L-78 showed a gradual increase in the percentage of apoptotic cells (from 6.85% at 0 min to 36.05% at 180 min) (Figure 5A). On the contrary, monocytes infected with ATCC 43816, showed lower percentages of apoptotic cells (18.15% at 180 min), but the percentage of necrotic cells increased early pi (18.30% at 15 min) and remained high (30.80% at 120 min pi) (Figure 5B). Unlike monocytes, human macrophages resisted infection with both *K. pneumoniae* strains, and much lower percentages of dead cells were recorded (< 10%). Specifically, few macrophages incubated with L-78 were apoptotic (1-2%) (Figure 5D). Macrophages incubated with ATCC 43816 showed a similar pattern of cell death, characterized by few apoptotic (< 2%) and necrotic (< 5%) cells (Figure 5E).

In parallel, supernatants of the aforementioned co-cultures were collected and analyzed by our ELISA. Gradually increasing concentrations of proTα(100-109), expressed as fold-increase, paralleled the increase of apoptotic cells from 30 to 180 min pi in the supernatants of L-78-infected monocytes (*p* = 0.0015). The concentration of proTα(100-109) in the supernatants of ATCC 43816-infected monocytes remained low and relatively constant up to 180 min pi, as less monocytes were driven to apoptosis (Figure 5C). On the contrary, as macrophages infected with either L-78 or ATCC 43816 remained alive, the quantity of proTα(100-109) detected in their culture supernatant was low (fold-increase < 3 for both bacterial strains, at all time points) (Figure 5F). Nevertheless, the differences observed in L-78-infected macrophages over time were statistically significant (*p* = 0.0324).

### Only L-78 was efficiently phagocytosed by human monocytes and macrophages

**Figure 6:**
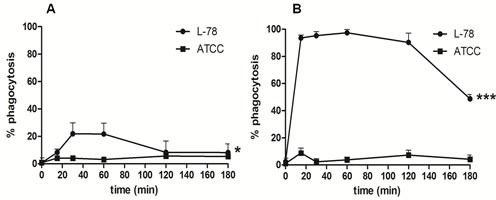
*K. pneumoniae* L-78 was phagocytosed by human macrophages and monocytes Human monocytes **A**. and *in vitro*-differentiated macrophages **B**., cultured on coverslips in DMEM supplemented with 1% FBS without antibiotics, were infected with CFSE-labeled L-78 (•) or ATCC 43816 (■) for 0-180 min, and analyzed by flow cytometry. Data are means ± SD from cells derived from 5 different donors. *, *p* = 0.0266 for L-78-infected monocytes; ***, *p* < 0.0001 for L-78-infected macrophages (one-way ANOVA).

Human monocytes and macrophages were incubated with the two *K. pneumoniae* strains labeled with 5(6)-carboxyfluorescein diacetate N-succinimidyl ester (CFSE) to visualize and monitor phagocytosis by flow cytometry. Monocytes incubated with L-78 showed a moderate gradual increase in bacterial phagocytosis up to 60 min pi, which was significantly reduced between 120-180 min. Conversely, monocytes co-incubated with ATCC 43816 for the same time did not phagocytose the bacteria, and minimum internalization was recorded (0.9-5.6%) (Figure 6A). In contrast to monocytes, macrophages rapidly and strongly phagocytosed L-78 up to 120 min pi and only at 180 min pi, L-78 phagocytosis was reduced by half. A very low percentage of ATCC 43816 was phagocytosed by macrophages (< 10% over 180 min) (Figure 6B).

Confocal microscopy confirmed the intracellular localization of L-78 in monocytes/macrophages, excluding the possibility of bacterial adherence on the cell surface. Analysis was conducted by examining coverslips on which monocytes and macrophages were co-incubated with CFSE-labeled bacteria for 30 min, a time point where high percentages of phagocytosis were recorded by flow cytometry (Figure 6). In monocytes and especially macrophages infected with L-78, their typical ellipsoidal and round nuclei were surrounded by green fluorescence, suggesting that L-78 was internalized and digested by both cell subpopulations. On the contrary, monocytes and macrophages infected with ATCC 43816 emitted blue fluorescence accumulated in their characteristic nuclei, and less green fluorescence, whereas non-phagocytosed bacteria appeared in the extracellular space.

### Serum levels of proTα(100-109) can predict mortality due to sepsis early pi

To validate the significance of our results, we generated a “moderate” model of infection, by infecting mice with 5×10^3^ CFU ATCC 43816 (LD_50_) [13]. In this case 50% of the animals were expected to die due to sepsis between 30-45 h pi. We were not able to generate a similar “moderate” mouse model of sepsis using higher inocula of L-78, as bacterial preparations corresponding to ≥10^10^ CFU/mL resulted in difficult to handle highly viscous preparations.

Mice that developed lethal septicemia and died had increased serum concentrations of proTα(100-109) as early as 3 h pi (Figure 1C, red circles). The differences were more pronounced at 6 and 12 h pi, where the levels of proTα(100-109) in serum of infected animals that died at 30-45 h were similar (> 5 ng/mL) to those of mice infected with the high (lethal) inoculum of ATCC 43816 (10^6^ CFU) (Figure 1B). On the contrary, mice that recovered infection and survived had lower proTα(100-109) levels in their serum at 6 and 12 h pi, and these resembled more the levels of the decapeptide in the serum of L-78-infected animals (< 5 ng/mL; Figure 1A). Overall, 4 infected mice with increased proTα(100-109) concentration in serum between 3-12 h pi died at 30-45 h, suggesting that very early determination of the levels of the decapeptide in their peripheral blood may predict sepsis-induced death.

## DISCUSSION

The necessity of introducing novel biomarkers for prognosis and early prediction of the outcome of septic patients is highly critical. In this study, we used the decapeptide proTα(100-109) as a biomarker to monitor the progression of sepsis in mice infected with *K. pneumoniae*. ProΤα(100-109) is the C-terminal fragment of proTα, a polypeptide with reported immunostimulatory activity [15], considered a danger signal or alarmin [10, 16–17].

We infected mice with two *K. pneumoniae* strains: the clinical isolate L-78 and the prototype ATCC 43816. Infection of mice with either strain, increased serum levels of proTα(100-109), but a differential pattern of increase over time was registered. In the L-78 model, the concentration of proTα(100-109) rose in a time-dependent manner after the onset of sepsis. Mice developed visible signs of bacteremia (lethargy, hunched posture, increased breathing rate and shiver), but as L-78 was phagocytosed and presumably successfully cleared, infection was resolved within 48 h. In contrast, in the 10^6^ CFU ATCC 43816 model, the levels of proTα(100-109) significantly increased early pi (at 3 h) and remained high until death of all animals at 15 h pi. In this case, the bacteria rapidly reached the spleen and rose exponentially, as confirmed by spleen cell cultures. These findings are in agreement with previous studies regarding the differential infectivity profiles of the two *K. pneumoniae* strains [13], as well as with the observed increase in proTα(100-109) serum levels in *S. pyogenes*-infected mice [11].

To verify that the levels of proTα(100-109) correlate with apoptosis, we assessed the cell death pattern of murine splenocytes following *K. pneumoniae* infection. L-78 infection induced mostly apoptosis, which was more apparent in splenic monocytes/macrophages, while infection with ATCC 43816 induced necrosis, predominantly at later time points pi. Our findings are in agreement with the study of Yang [18], who elegantly demonstrated that *in vitro* infection of cells with whole live *K. pneumoniae*, induced multiple (apoptotic and necrotic) cell death pathways concurrently. Therefore, we suggest cellular apoptosis and necrosis as causative elements for the presence of proTα(100-109) and probably intact proTα in blood serum of *K. pneumoniae*-infected mice, respectively.

Activation of apoptotic pathways directly relates to alterations in procaspase-3/caspase-3 levels, and results in degradation of cellular components [19], including cleavage of proTα [6]. Indeed, in L-78 infection, the levels of procaspase-3 were significantly reduced at the early stages pi indicating activation of caspase-3, whereas in the ATCC 43816 model, procaspase-3 levels decreased less. In agreement with the study of Lin [20], who also showed that activated caspase-3 was significantly elevated in murine liver lysates upon *K. pneumoniae* infection, our results suggest that L-78 infection massively induces caspase-3-mediated apoptosis at early stages pi *in vivo*.

To extend the aforementioned host-pathogen interactions in man, we exposed human monocytes/macrophages to the two *K. pneumoniae* strains. Monocytes infected with L-78 died mainly by apoptosis, whereas those infected with ATCC 43816 mostly by necrosis. As also shown by others [21], monocytes were more susceptible to death compared to macrophages, which were highly resistant. When we quantified the levels of proTα(100-109) in infected monocyte and macrophage culture supernatants, we recorded significantly elevated proTα(100-109) concentration in supernatants of L-78-infected monocytes. In agreement with the *in vivo* results, high proTα(100-109) levels corresponded to increased percentages of apoptotic cells. Confocal microscopy confirmed that L-78 was mostly internalized early pi, whereas at the same time point, ATCC 43816 was present, almost exclusively, extracellularly. This result is in line with previous data showing that L-78 was efficiently phagocytosed by the murine macrophage cell line J774A.1 [13].

Finally, we tested the predictive ability of our assay in a “moderate” model of sepsis induced by *K. pneunoniae* ATCC 43816 strain. We infected mice with an inoculum corresponding to the LD_50_ of ATCC 43816 [13] and quantified serum levels of proTα(100-109) over 48 h. High levels of the decapeptide in the serum of animals which finally died due to sepsis were determined as early as 3 h pi, suggesting that proTα(100-109) could serve as an early surrogate biomarker for sepsis outcome.

Although experiments in additional infectious models are warranted, to our knowledge this is the first report showing that the levels of proTα(100-109) can serve as a biomarker for monitoring the outcome of bacteria (herein *K. pneumoniae*)-induced sepsis. Quantification of proTα(100-109) in serum is advantageous compared to HMGB1, thoroughly studied in sepsis. Firstly, generation of proTα(100-109) is a very early apoptotic event (occurring within the first 2 h) [6] and so proTα(100-109) can serve as a very early sepsis biomarker, in contrast to HMGB1 that is a late sepsis mediator. Secondly, although the exact mechanism of proTα(100-109) release from cells is yet unknown, the presence of proTα(100-109) extracellularly is strongly associated with massive cell apoptosis induced by an infectious agent. Preliminary results in humans show that serum proTα(100-109) levels increase in septic patients, but not in cases of “sterile inflammation”, developed for instance in patients with autoinflammatory diseases (P. Samara et al., unpublished data). In contrast, increased levels of HMGB1 have been reported in several non-infectious conditions [22].

Based on the results presented here, we propose a realistic scenario on the significance of extracellular proTα(100-109) quantification relevant to the diverse mechanisms of sepsis induced by the two *K. pneumoniae* strains (Figure 8): L-78 is phagocytosed by innate immune cells, caspase-3 is activated and cells are driven to apoptosis. Apoptotic cells gradually excrete proTα(100-109), resulting in progressive stimulation of immune responses. Therefore, no toxicity for instance due to excess cytokine secretion occurs, and this concomitantly with L-78 clearance by monocytes/macrophages, leads to recovery from sepsis. On the contrary, infection with the non-phagocytosed ATCC 43816 leads to massive cell necrosis, abrupt release of high concentrations of alarmins, including proTα, excess immune system activation, irreversible septic shock, and death. In any case, determination of proTα(100-109) at the early phase of sepsis suggests its potential utility as an early sepsis biomarker. Clinical studies in humans, currently in progress in our laboratory, will further evaluate the usefulness of serum proTα(100-109) levels as a surrogate biomarker in monitoring bacterial sepsis.

**Figure 7:**
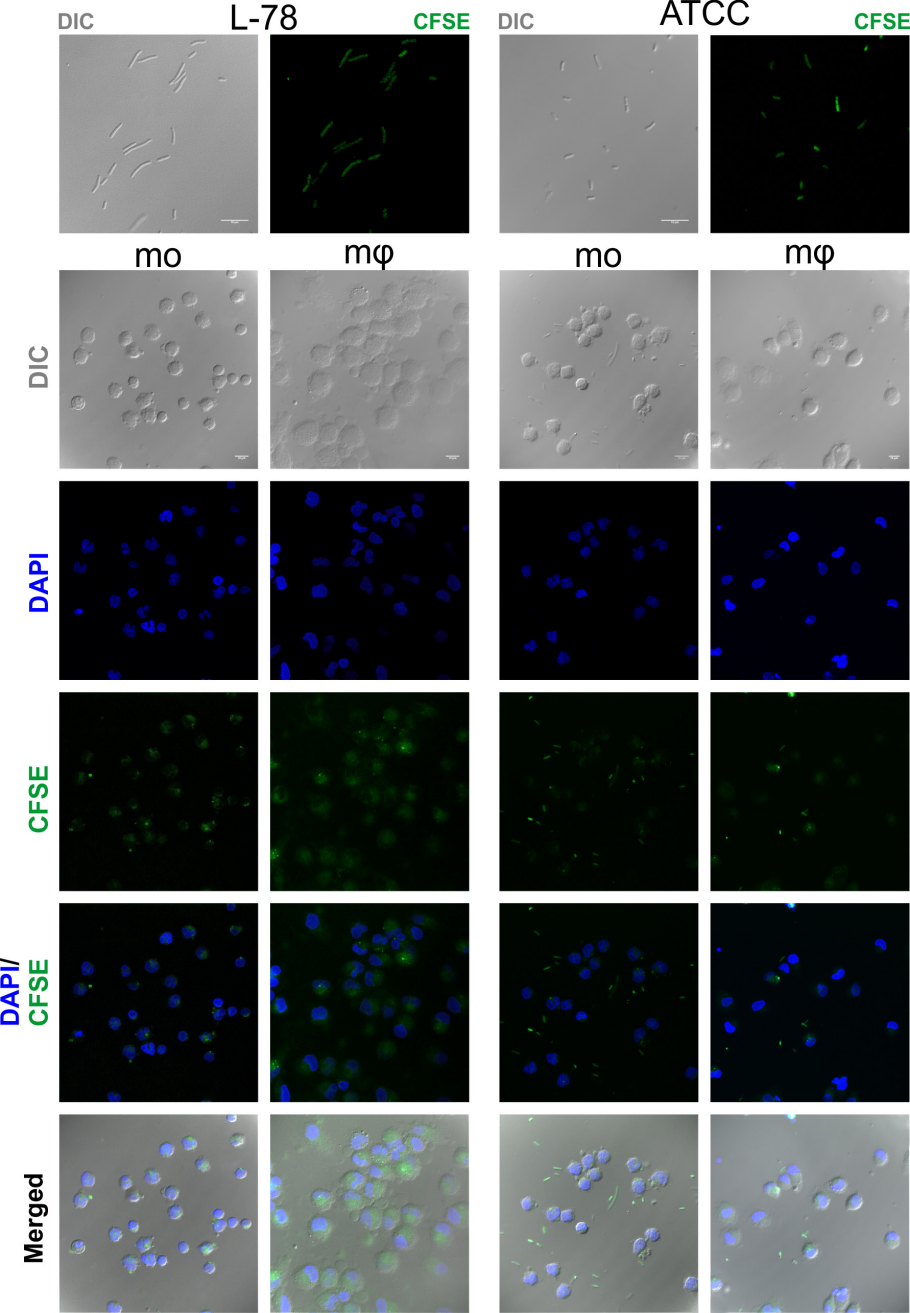
Intracellular localization of *K. pneumoniae* L-78 Cells from the same donors treated as described in legend of Figure 6, were examined using a confocal microscope. ATCC 43816 and L-78 were stained green with CFSE. Nuclei were stained blue with DAPI. The frames show representative optical fields of the labeled bacteria L-78 and ATCC 43816 (first row), and of monocytes (mo) and macrophages (mφ) incubated with L-78 and ATCC 43816 for 30 min. Nomarski images represent the average projection of 6 to 20 optical sections taken at 0.50 μm intervals on the z axis, except the L-78 bacteria (1 optical section). DAPI and CFSE images represent the maximum projection of the same above stacks. Scale bars, 10 μm. Images from one representative experiment out of 5 performed are shown.

**Figure 8:**
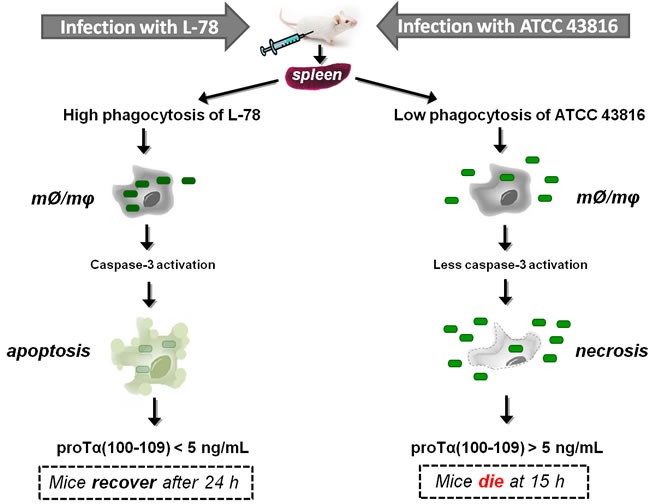
Proposed scenario supporting the eventual use of proTα(100-109) as an early surrogate biomarker of sepsis *Klebsiella pneumoniae* strains L-78 (left) and ATCC 43816 (right) intraperitoneally injected in mice enter the bloodstream through the microcirculation of the peritoneum, infect internal organs, e.g. spleen, and multiply. In spleen, monocytes/macrophages (mØ/mφ) phagocytose L-78 (green rectangles) early post-infection (3 h) and caspase-3 levels are increased, driving cells to apoptosis. In apoptotic cells, proTα is cleaved by activated caspase-3 and the decapeptide proTα(100-109) is gradually exocytosed. Finally, L-78 is cleared; mice control the infection (at ∼24 h) and recover. On the contrary, ATCC 43816 is minimally phagocytosed by spleen monocytes/macrophages (mØ/mφ), thereby remaining in the extracellular space where they rapidly multiply. In this case, monocytes/macrophages are led to necrosis (12 h) and caspase-3 is less activated. High levels of DAMPs are extracellularly released, the bacteria are not cleared, sepsis and multiple organ failure occur, and by 15 h all mice die.

## MATERIALS AND METHODS

### Ethical issues

Experiments involving animals were performed in strict accordance with the guidelines of the European Convention for the Protection of Vertebrate Animals Used for Experimental and Other Scientific Purposes. All animal experiments were approved by the Animal Care and Use Committee of the Hellenic Pasteur Institute and by National Authorities (Veterinary Section of the Greek Republic). The study involving human samples was approved by the Ethical Committee of the 2^nd^ Peripheral Blood Transfusion Unit and Hemophilia Center, “Laikon” General Hospital, Athens, Greece and informed consent was obtained from all subjects. The study was carried out in accordance with the Declaration of Helsinki, as revised in 2013.

### Bacterial strains and culture conditions

Two strains of the Gram-negative bacillus *K. pneumoniae* of different virulence were used. *K. pneumoniae* KPC-Kp ST258 (L-78) is a clinical isolate derived from patients with bacteremia in Greek hospitals during 2009-2011 and its identity has been previously confirmed by standard techniques [13]. *K*. *pneumoniae* ATCC 43816 is a K2 strain used in animal models of infection. Both bacterial strains were stored at −80°C in medium containing 20% glycerol and were grown in freshly prepared Luria-Bertani (LB) agar (Sigma-Aldrich, St Louis, MO). Bacteria were incubated at 37°C in ambient air in order to reach the log phase of growth. Bacterial growth was spectrophotometrically monitored and adjusted to an initial optical density (OD_580nm_) of 0.3. Prior animal infection, bacterial preparations were adjusted to 10^9^ CFU/mL for the L-78 strain and 5×10^4^ or 10^7^ CFU/mL for the ATCC 43816 strain.

### In *silico* analysis of K. *pneumoniae* and mouse genomes

We retrieved *K. pneumoniae* protein sequence data available in publicly accessible sequence databases. In particular, data regarding *K. pneumoniae* complete genomes were retrieved from the NCBI website (http://www.ncbi.nlm.nih.gov/genome/genomes/815; accessed 9 June 2016). In total, the predicted protein complements for 58 genomes were retrieved. In addition, we retrieved protein sequence sets from the same source for 7 yet incomplete genomes for *K. pneumoniae* strains which are related to the strains studied in this work for completeness. A list of the *K. pneumoniae* protein datasets used in this work is provided in Supplementary Table S1. Proteins encoded in the mouse genome were downloaded from ENSEMBL (accessed 8 June 2016; build GRCm38.p4) [23]. For representing the proTα(100-109)-like peptides, we used the same sequence set and alignments described in [11].

### In *vivo* models of sepsis

Female ICR (CD-1) mice aged 6 to 8 weeks with a weight of 25-30 g were maintained in the Department of Animal Models for Biomedical Research of the Hellenic Pasteur Institute under pathogen-free conditions. Mice were ip injected with 10^8^ CFU per mouse of the L-78 strain (*n* = 18), 5×10^3^ or 10^6^ CFU/mouse of the ATCC 43816 strain (*n* = 10 and *n* = 16, respectively) and monitored every 2 h for clinical signs of bacteremia (lethargy, hunched posture, increased breathing rate or shiver). Blood was collected by retro-orbital sinus puncture using a glass Pasteur pipette before infection (control, 0 h), at 3, 6, 12, 24, and 48 h pi for L-78 and 5×10^3^ ATCC 43816 groups, and at 3, 6, 12, and 15 h pi for 10^6^ ATCC 43816 group. At each time point, two animals from the L-78 and 10^6^ ATCC 43816 groups were euthanized by cervical dislocation. Spleens were aseptically removed, weighed and homogenized in sterile Dulbecco`s phosphate-buffered saline (DPBS; Lonza, Cologne, Germany). An aliquot of the homogenate was analyzed by flow cytometry and Western blotting, while the remaining homogenate was diluted and plated onto LB agar to determine the number of viable microorganism CFUs.

### Isolation of peripheral blood mononuclear cells (PBMCs)

Human buffy coat preparations were used as a source of peripheral blood mononuclear cells (PBMCs) isolated by centrifugation over Ficoll-Histopaque (Sigma-Aldrich) density gradient. PBMCs were resuspended (10-15×10^6^/mL) in DMEM, supplemented with 10% heat-inactivated fetal bovine serum (FBS), 10 mM Hepes, 5 μg/mL gentamycin and 10^2^ U/mL penicillin/streptomycin (all from Lonza). Highly enriched monocytes (> 85% CD14+ as verified by flow cytometry following staining with FITC anti-human CD14 antibody [BioLegend, London, UK]) were obtained from PBMCs by plastic adherence for 2 h at 37°C in a humidified 5% CO_2_ incubator. Macrophages were differentiated from monocytes by incubation with 100 ng/mL recombinant human granulocyte macrophage colony-stimulating factor (R&D Systems GmbH, Wiesbaden-Nordenstadt, Germany) for 5 days [24]. Differentiation was confirmed by flow cytometry, by staining macrophages with APC-Cy7 anti-human CD206 antibody (BioLegend). Both monocytes and macrophages were *in vitro* infected with L-78 or ATCC 43816. The levels of proTα(100-109) were quantified in supernatants by ELISA, whereas the type of induced cell death and their phagocytic ability were analyzed by flow cytometry and confocal microscopy.

### Enzyme-linked immunosorbent assay (ELISA) for proTα(100-109)

Blood samples from each mouse were individually treated as previously described [11]. Precipitated mouse sera or supernatants from *K. pneumoniae*-infected human monocytes or macrophages were analyzed in duplicates by our in-house developed ELISA, and the quantity of proTα(100-109) was expressed in ng/mL using the linear part of the standard curve [11].

### Evaluation of cell apoptosis

To evaluate morphological changes in splenocytes of *Klebsiella*-infected mice, as well as in *Klebsiella*-infected human monocytes/macrophages, cells were stained with Annexin V and PI [25]. Annexin V binds to phosphatidylserine exposed on the outer leaflet of the cell membrane of apoptotic cells, while PI is a DNA-binding dye, which enters cells when their membranes are ruptured. For cell labeling, the FITC Annexin V apoptosis kit with PI (BioLegend) was used and staining was performed as recommended by the manufacturer. For adherent cells, i.e., human monocytes and macrophages, detachment *via* 1X trypsin/EDTA solution (Lonza) was used and viability was > 90% as confirmed by Trypan Blue exclusion [26]. Cells were washed twice with PBS and resuspended in Annexin V binding buffer at a concentration of 10^6^ cells/mL. One hundred μL of cell suspension were transferred in FACS tubes, where 5 μL of FITC Annexin V and 10 μL of PI were added. Cells were gently vortexed and incubated for 15 min at room temperature in the dark. Four hundred μL of Annexin V binding buffer were added to each tube and cells were analyzed by a FACSCanto II flow cytometer (Becton-Dickinson [BD] Biosciences, Erembodegem, Belgium) equipped with FACSDiva software (BD Biosciences). Monocytes/macrophages from mouse spleens were gated using phycoerythrin anti-mouse CD11b antibody (BioLegend) and analyzed separately.

### Western blotting

Splenocytes were lysed in lysis buffer (50 mM Tris-HCl, 150 mM NaCl, 2 mM EDTA, pH 7.6, 1% Triton X-100 [v/v]) supplemented with Complete^®^ protease inhibitor cocktail (Roche Life Science, Manheim, Germany) for 30 min on ice. Cell lysates were centrifuged at 10,000g for 10 min at 4°C and protein concentration in the supernatants was determined using the Bradford protein assay (SERVA Electrophoresis GmbH, Heidelberg, Germany). In parallel, HeLa cells (ATCC CCL-2), led *in vitro* to apoptosis (4 h incubation with 5 ng/mL TNF-α and 1 μg/mL emetine [both from Sigma-Aldrich]) or necrosis (4 h incubation with 100 μg/mL doxorubicin [Sigma-Aldrich]), were used as controls [6]. Equal amounts of protein (20 μg/lane) were separated by SDS-PAGE on 15% (w/v) gels and transferred onto nitrocellulose membranes. The blots were blocked in a solution of 5% (w/v) nonfat dry milk in Tris-buffered saline containing Tween-20 (TBS-T; 20 mM Tris-HCl, pH 7.5, 137 mM NaCl, 0.05% [v/v] Tween-20) for 1 h at room temperature, followed by overnight incubation at 4°C with primary antibodies against procaspase-3 (1:500 in TBS-T; ab44976, Abcam, Cambridge, UK) or β-actin (1:2500; Sigma-Aldrich). After washing with TBS-T (4×5 min), blots were incubated with the appropriate HRP-conjugated secondary antibody (1:5000 in TBS-T containing 1% [w/v] nonfat dry milk) for 1 h at room temperature, washed again with TBS-T (4×5 min), developed using the enhanced chemiluminescence reagent (ECL reagent; Amersham Biosciences, Buckinghamshire, UK) and quantified by densitometry (Gel Analyzer v.1.0, Biosure, Athens, Greece). All densitometric values were normalized against β-actin levels. Procaspase-3 levels in control samples were set at one, and the respective values of treated samples are expressed as fold-induction over control samples.

### Analysis of phagocytosis by flow cytometry

The two strains of *K. pneumoniae* were labeled with the fluorescent dye CFSE (Sigma-Aldrich) according to Sokolovska [27]. Human monocytes or macrophages were incubated with the bacteria following the protocol described in [27] with slight modifications. In brief, 4.5-5×10^5^ monocytes or macrophages were plated per well of a 6-well culture plate (Greiner Bio-One GmbH, Frickenhausen, Germany) in 2 mL DMEM-1% FBS-without antibiotics (w/a). The medium was replaced with 1.5 mL ice-cold DMEM-1% FBS-w/a containing the CFSE-labeled bacterial particles at a multiplicity of infection (MOI) of 25. Plates were centrifuged (100g, 4°C, 5 min) to allow bacteria to settle onto the cells and incubated at 37°C for 15, 30, 60, 120, and 180 min. Wells placed on ice (4°C) were used as controls. Phagocytosis was stopped by adding 1.5 mL of ice-cold DPBS containing 5 mM EDTA (Lonza) to each well. Cells were washed twice with DPBS/EDTA, once with DPBS and were detached using 1X trypsin/EDTA solution. Cells were then centrifuged, resuspended in 400 μL DPBS, transferred to FACS tubes placed on ice, and immediately analyzed by flow cytometry. The results are presented as percentage of monocytes or macrophages bearing internalized bacteria (% phagocytosis) *versus* time.

### Analysis of phagocytosis by confocal microscopy

Human monocytes or macrophages were grown on sterilized 12×12 mm coverslips (Sigma-Aldrich) precoated with poly-L-lysine (50 μg/mL for 1 h at 37°C; Sigma-Aldrich) and placed in 6-well culture plates at 37°C in a humidified 5% CO_2_ incubator. Cells were infected with the two *Klebsiella* strains for 30 min, as described in the previous paragraph, fixed with 4% paraformaldehyde diluted in DPBS for 15 min at room temperature and washed three times with DPBS. Each coverslip was placed on a single slide containing mounting medium with DAPI (Sigma-Aldrich) and protected from light. Specimens were examined on a multiphoton confocal microscope Leica TCS SP8 MP (Wetzlar, Germany) equipped with an Argon laser (excitation lines at 458, 476, 488, 496, and 514nm) and an IR MaiTai DeepSee Ti:Sapphire laser (Spectra-Physics, Santa Clara, CA, USA) for multiphoton applications. Images were acquired with the spectral detector of the microscope using appropriate emission wavelength ranges; CFSE (green) at 500-550nm and DAPI (blue) at 420-500nm. Acquisition was performed with the LAS X software (Leica Microsystems CMS GmbH, Wetzlar, Germany) using the same parameters (laser power, gain, pinhole, speed and analysis) for all specimens. Images were acquired as stacks of 6 to 20 optical sections with a Z-step of 0.50 μm. Average or Maximum Projections of the stacks were created with the LAS X software and minor adjustments to contrast, brightness and levels with Adobe Photoshop CC (Adobe Systems Inc., San Jose, CA, USA) for better representation.

### Statistical analysis

Each *in vitro* and *in vivo* experiment was conducted at least three times. All data were analyzed using GraphPad Prism 5 software (GraphPad Software, San Diego, CA, USA). Results are expressed as means ± standard deviation (SD). For statistics, we used Student's *t*-test and one-way analysis of variance (ANOVA) followed, where indicated, by Dunnett's test. *P*-values < 0.05 were considered statistically significant.

## SUPPLEMENTARY MATERIAL FIGURES AND TABLE



## Abbreviations

CFU: colony-forming unit
DAMP: damage-associated molecular pattern
HMGB1: high-mobility group box 1 protein
*K. pneumoniae*: *Klebsiella pneumoniae*
PI: propidium iodide
proTα: prothymosin alpha
*S. pyogenes*: *Streptococcus pyogenes*
