# Supplementary material for: A fragment of the alarmin prothymosin α as a novel biomarker in murine models of bacteria-induced sepsis
Source: Oncotarget. 2017 May 24;8(30):48635–49. doi: 10.18632/oncotarget.18149 (PMC5564713; doi:10.18632/oncotarget.18149)
Supplement: Supplementary file 1 [file oncotarget-08-48635-s001.pdf]

# A fragment of the alarmin prothymosin α as a novel biomarker in murine models of bacteria-induced sepsis

## Supplementary Material

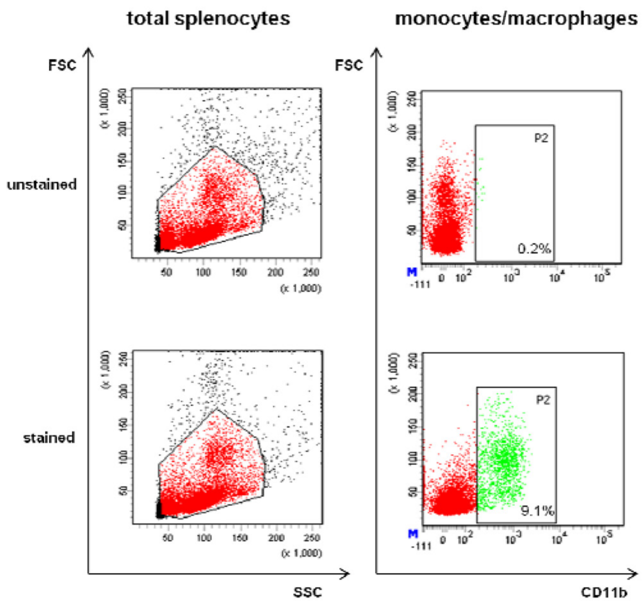


**Supplementary Figure S1: Flow cytometry analysis of murine spleen cells, isolated from CD-1 mice.** Total splenocytes (left) and monocytes/macrophages (right) unstained (first row) and stained with anti-mouse CD11b (second row) are gated. Representative dot plots from an uninfected mouse at 0 h are shown.

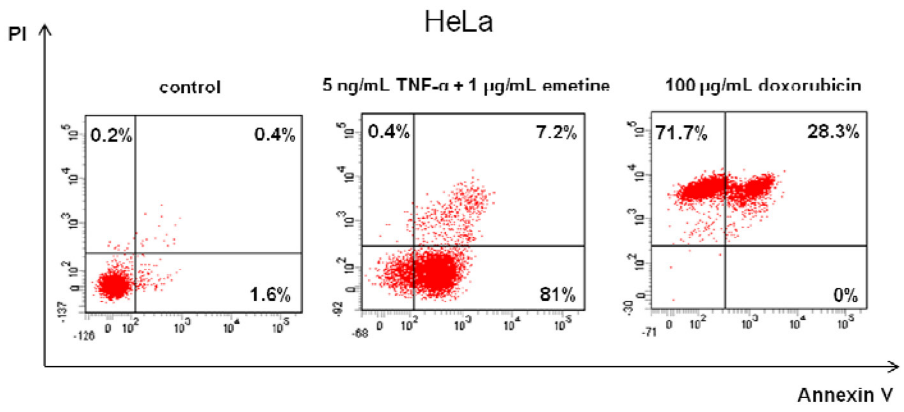


**Supplementray Figure S2: HeLa cells in vitro driven to death were analyzed by flow cytometry.** Untreated HeLa (control) and HeLa cells treated with 5 ng/mL TNF-α and 1 µg/mL emetine or 100 µg/mL doxorubicin for 4 h, were stained using Annnexin V/PI and immediately analyzed. Percentages of cells led to apoptosis are shown in the low right quadrant. Percentages of necrotic cells are shown in the upper quadrants. HeLa cells treated with TNF-α and emetine were mostly driven to apoptosis (81%), whereas all cells treated with doxorubicin were necrotic (100%).

**Supplementary Table S1: List of Klebsiella pneumoniae genomes used in this study.** The dataset, containing a total of 350,269 sequences was scanned for the presence of proTα(100-109)-like peptides.

| Klebsiella pneumoniae strain | Number of protein coding genes | RefSeq assembly accession number |
|---|---|---|
| HS11286 | 5779 | GCF_000240185.1 |
| NTUH-K2044 | 5182 | GCF_000009885.1 |
| ATCC 700721; MGH 78578 | 5453 | GCF_000016305.1 |
| 342 | 5369 | GCF_000019565.1 |
| KCTC 2242 | 5218 | GCF_000220485.1 |
| KPNIH10 | 5709 | GCF_000281435.2 |
| KPNIH1 | 5712 | GCF_000281535.2 |
| 1084 | 4995 | GCF_000294365.1 |
| ATCC BAA-2146 | 5707 | GCF_000364385.3 |
| 500_1420 | 5470 | GCF_000406765.2 |
| UHKPC33 | 5582 | GCF_000417085.2 |
| DMC1097 | 5609 | GCF_000417225.2 |
| UHKPC07 | 5464 | GCF_000417265.2 |
| JM45 | 5389 | GCF_000445405.1 |
| KP-1 | 5151 | GCF_000465975.2 |
| CG43 | 4918 | GCF_000474015.1 |
| Kp13 | 5488 | GCF_000512165.1 |
| 30684/NJST258_2 | 5326 | GCF_000597905.1 |
| 30660/NJST258_1 | 5420 | GCF_000598005.1 |
| KPNIH27 | 5948 | GCF_000695935.1 |
| KPNIH24 | 5657 | GCF_000714675.1 |
| KPR0928 | 5367 | GCF_000717515.1 |
| PittNDM01 | 5515 | GCF_000733255.1 |
| blaNDM-1 | 5379 | GCF_000739495.1 |
| ATCC 43816 KPPR1 | 5074 | GCF_000742755.1 |
| PMK1 | 5675 | GCF_000764615.1 |
| KPNIH33 | 5545 | GCF_000775375.1 |
| KPNIH32 | 5742 | GCF_000775395.1 |
| XH209 | 4888 | GCF_000775955.1 |
| KPNIH29 | 5296 | GCF_000784945.1 |
| KPNIH30 | 5420 | GCF_000784985.1 |
| KPNIH31 | 5250 | GCF_000785005.1 |
| 32192 | 5488 | GCF_000807395.2 |
| HK787 | 5091 | GCF_000813205.1 |
| 34618 | 5559 | GCF_000814305.1 |
| 1158 | 5028 | GCF_000814805.1 |
| Kp52.145 | 5320 | GCF_000968155.1 |
| 234-12 | 5391 | GCF_000981845.1 |
| CAV1392 | 5447 | GCF_001022035.1 |
| CAV1344 | 5667 | GCF_001022175.1 |
| CAV1596 | 5507 | GCF_001022235.1 |

| | | |
|---|---|---|
| HKUOPLC | 4770 | GCF_001280925.1 |
| KP617 | 5415 | GCF_001307175.1 |
| MS6671 | 5743 | GCF_001455995.1 |
| KpN01 | 5504 | GCF_001456055.3 |
| KpN06 | 5476 | GCF_001456095.3 |
| CAV1193 | 5735 | GCF_001456135.1 |
| J1 | 5175 | GCF_001482345.1 |
| NUHL24835 | 5292 | GCF_001521895.1 |
| RJF999 | 5372 | GCF_001529935.1 |
| RJF293 | 5143 | GCF_001530015.1 |
| YH43 | 5001 | GCF_001548315.1 |
| TGH8 | 5210 | GCF_001611055.1 |
| TGH10 | 5155 | GCF_001611095.1 |
| SKGH01 | 5777 | GCF_001644765.1 |
| W14 | 5212 | GCF_001646625.1 |
| AATZP | 5305 | GCF_001648215.1 |
| KP5-1 | 4918 | GCA_000714635.1 ** |
| ST258 * | 5615 | GCF_001238585.1 |
| ST258-490 * | 5616 | GCF_000283455.1 |
| ST258-K26BO * | 5268 | GCF_000313465.1 |
| ST258-K28BO * | 5447 | GCF_000313365.1 |
| ST258_FL * | 5708 | GCF_001008725.1 |
| KPC45 * | 5276 | GCF_001645745.1 |
| 45T1-2A * | 4941 | GCF_001373075.1 |

*denotes strains with yet incomplete genomes
**for K. pneumoniae KP5-1, a RefSeq assembly was not available and protein sequences were derived based on the existing GenBank entry
